# Landscape transcriptomic analysis of bovine follicular cells during key phases of ovarian follicular development

**DOI:** 10.1186/s40659-024-00558-2

**Published:** 2024-10-28

**Authors:** Henry David Mogollón García, Rodrigo de Andrade Ferrazza, Julian Camilo Ochoa, Flávia Florencio de Athayde, Pedro Marcus Pereira Vidigal, Milo Wiltbank, John Patrick Kastelic, Roberto Sartori, João Carlos Pinheiro Ferreira

**Affiliations:** 1 Department of Veterinary Surgery and Animal Reproduction, School of Veterinary Medicine and Animal Science, São Paulo State University (UNESP), Rua Prof. Doutor Walter Mauricio Correa, s/n, Botucatu, São Paulo 18618-681 Brazil; 2 Department of Genetic, Evolution, Microbiology and Immunology. Biology Institute, Campinas State University, Campinas, São Paulo Brazil; 3 Computational Systems Biology Laboratory (CSBL), Institut Pasteur, University of São Paulo (USP), São Paulo, Brazil; 4 Department of Animal Science, Londrina State University, Londrina, Paraná Brazil; 5 Department of Animal Production and Health, School of Veterinary Medicine, São Paulo State University (UNESP), Araçatuba, São Paulo Brazil; 6 Center of Biological Sciences, Viçosa Federal University, Viçosa, Minas Gerais Brazil; 7 Department of Animal & Dairy Sciences, University of Wisconsin-Madison, Madison, USA; 8 Faculty of Veterinary Medicine, University of Calgary, Calgary, AB Canada; 9 Department of Animal Science, Luiz de Queiroz College of Agriculture (ESALQ), University of São Paulo, Piracicaba, São Paulo Brazil

**Keywords:** Holstein cows, Genes, Deviation, Steroidogenesis

## Abstract

**Background:**

There are many gaps in our understanding of the mechanisms involved in ovarian follicular development in cattle, particularly regarding follicular deviation, acquisition of ovulatory capacity, and preovulatory changes. Molecular evaluations of ovarian follicular cells during follicular development in cattle, especially serial transcriptomic analyses across key growth phases, have not been reported. This study aims to address this gap by analyzing gene expression using RNA-seq in granulosa and antral cells recovered from ovarian follicular fluid during critical phases of ovarian follicular development in Holstein cows.

**Results:**

Integrated analysis of gene ontology (GO), gene set enrichment (GSEA), protein–protein interaction (PPI), and gene topology identified that differentially expressed genes (DEGs) in the largest ovarian follicles at deviation (Dev) were primarily involved in FSH-negative feedback, steroidogenesis, cell proliferation, apoptosis, and the prevention of early follicle rupture. In contrast, DEGs in the second largest follicles (DevF2) were mainly related to loss of cell viability, apoptosis, and immune cell invasion. In the dominant (PostDev) and preovulatory (PreOv) follicles, DEGs were associated with vascular changes and inflammatory responses.

**Conclusions:**

The transcriptome of ovarian follicular fluid cells had a predominance of granulosa cells in the dominant follicle at deviation, with upregulation of genes involved in cell viability, steroidogenesis, and apoptosis prevention, whereas in the non-selected follicle there was upregulation of cell death-related transcripts. Immune cell transcripts increased significantly after deviation, particularly in preovulatory follicles, indicating strong intrafollicular chemotactic activity. We inferred that immune cell invasion occurred despite an intact basal lamina, contributing to follicular maturation.

**Graphical Abstract:**

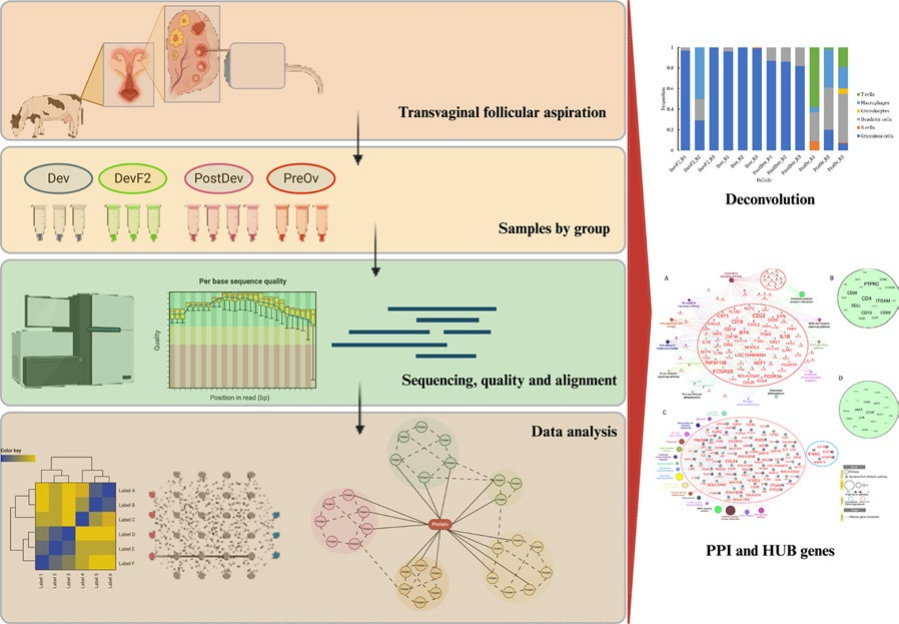

**Supplementary Information:**

The online version contains supplementary material available at 10.1186/s40659-024-00558-2.

## Background

Antral follicular development in cattle follows a wave-like pattern, usually two or three follicular waves [1]. In *Bos taurus*, emergence of an ovarian follicular wave involves synchronous growth of multiple small antral follicles [2]. Typically, when the largest follicle reaches a diameter of ~ 8.5 mm, deviation occurs, characterized by a reduction in growth rate of all follicles except the dominant [3]. From this moment forward, this follicle is defined as the dominant follicle, whereas all other follicles are defined as subordinates [3–5].

The bovine ovary has numerous oocytes plus somatic cells, including stromal, theca, and granulosa cells (GCs) [1]. Coordinated by neuroendocrine, paracrine, and autocrine factors, these cells interact and rearrange to form ovarian follicles. Throughout their development, ovarian follicles undergo morphological changes driven by a high cell proliferation rate, increasing diameter and antrum development [2]. Investigating molecular mechanisms behind these processes in cattle has been a focal point in ovarian physiology studies. Specific roles of certain agents, such as the luteinizing hormone/choriogonadotropin receptor (LHCGR), members of the fibroblast growth factor family (FGFs) [6], and insulin-like growth factors (IGFs) and their binding proteins (IGFBPs), have been elucidated in relation to follicle cell proliferation and differentiation [7]. However, it is increasingly apparent that follicular development is intricate, multifactorial, and involves numerous yet undiscovered agents.

In the last decade, studies incorporating transcriptomic analyses have enabled identification of a myriad of differentially expressed genes in granulosa cells of ewes [6], sows [7], and mares [8] related to coordination of ovarian follicular development. In cows, comparing the transcriptome of GCs from follicles at pre-deviation and deviation stages documented increased expression of genes associated with steroidogenesis [9]. In post-deviation follicles (> 10 mm), comparing transcriptomes of granulosa, theca, and small and large luteal cells revealed expression of genes involved in mitosis, DNA replication, and cell structure and repair [10]. Another analysis of follicles defined as small (3–5 mm) versus large (> 9 mm) detected expression of four genes (*MGARP, GLDC, CHST8, GPX3*) that were considered potential markers of GCs [11]. Transcriptomic analysis of large follicles (> 10 mm) revealed *STAT*, a mediator of the interleukin 12 (IL12) immune response [12, 13] and *XBP1*, an inducer of chaperones expression [14], as upstream genes, whereas the *MGEA5* gene, involved in O-linked glycosylation of proteins, was considered upstream in small follicles (< 5 mm). In the same study, functional enrichment of large follicles was associated with processes of axonal guidance, immune signaling, and cell rearrangement [15].

It is noteworthy that none of the previous transcriptomic studies involved serial monitoring of follicular development and consecutive samples collected at specific developmental phases. Thus, the aim of the present study was to analyze the transcriptome of granulosa and antral cells recovered from follicular fluid of Holstein cows at key phases of ovarian follicular development.

## Materials and methods

### Ethics approval

This study was performed at the School of Veterinary Medicine and Animal Science—UNESP and was approved by the Ethics Committee on Animal Use (CEUA FMVZ-Unesp: Permit number 86/2013).

### Animals and experimental design

The experiment was conducted at the Lageado Experimental Farm, School of Veterinary Medicine and Animal Science, Sao Paulo State University (UNESP), Botucatu, SP, Brazil. Eighteen multiparous nonlactating Holstein cows, 3–7 years old, with a body condition score ~ 3.5 (1–5 scale) [16] were used. Cows belonged to the School of Veterinary Medicine and Animal Science herd and, after the study, were returned to their original herd.

During the experiment, all cows had free access to pasture shade and stock waterers for ad libitum water. Nutritional management included access to pasture, supplemented with a total mixed ration. The study was conducted during the winter to avoid effects of heat stress.

Ovarian follicular development was synchronized using an Ovsynch protocol [17], combined with an intravaginal progesterone (P4)-releasing device (Sincrogest, Ourofino, Cravinhos, Brazil). Only 10 cows ovulated following the protocol and were used in the initial part of the study.

Following the post-synchronization ovulation, transrectal ovarian ultrasonography was conducted every 12 h (MyLab30 equipped with a 7.5 MHz linear-array transducer, Esaote, Genova, Italy) to monitor development of a new follicular wave. Once the largest pre-deviation follicle reached a diameter of ~ 7.0 mm (PreDev) [4], its follicular fluid was selectively aspirated and all remaining follicles (> 2 mm) were ablated to induce emergence of a new follicular wave. Subsequently, follicular growth was monitored every 12 h, and when the largest follicle reached a diameter of ~ 8.5 mm, the follicular fluid of both the largest (Dev) and the second largest (DevF2) follicles was individually aspirated, with all remaining follicles (> 2 mm) ablated. Following this, follicular development was once again monitored every 12 h, and when the largest follicle reached a diameter compatible with acquisition of ovulatory capacity (~ 12 mm; PostDev), its fluid was aspirated. After this, all 18 cows underwent synchronization using the previously described protocol. On the ninth day of the protocol, only 13 cows had a single follicle (≥ 12 mm) and received a second GnRH treatment (50 µg; Gestran Plus, São Paulo, Brazil); 24 h later, these follicles were aspirated (PreOv).

### In vivo transvaginal follicular aspiration

Transvaginal follicular aspiration was performed as described [18]. Briefly, follicles were aspirated using an ultrasound device (Mindray DP-3300 Vet, Mindray Bio-Medical Electronics Co. Ltd, Sheuzheu, China) equipped with a micro-convex 5 MHz transducer coupled to a needle guide system (WTA, Watan-Applied Technology, Cravinhos, Brazil) and connected to an aspiration line (WTA) and 20G disposable needle (WTA). The differential pressure applied to recover the follicular fluid was created using a 10 mL syringe (Descarpack, Sao Paulo, Brazil). Before aspiration sessions, caudal epidural anesthesia was induced (5 mL of 2% lidocaine; Lidovet, Bravet, Rio de Janeiro, Brazil). Then, the aspiration guide was inserted into the vagina and the ovary was positioned (via transrectal manipulation) against the vaginal wall over the transducer face so that the targeted follicle was transected by the built-in line on the ultrasound image representing the projected needle path. The needle tip was then advanced to the center of the targeted follicle and its follicular fluid aspirated. Only follicular fluid samples without macroscopic blood contamination were retained for subsequent analyses.

After collection, follicular fluid samples were centrifuged at 2000 × *g* for 10 min at 4 °C, and the supernatant and pellet (cells) were separated and stored at − 80 °C. Before storage, GCs were placed in cryotubes containing 350 µL of Lysis Buffer (RNEasy).

### RNA extraction and sequencing

Concentrations of P4 and estradiol (E2) in follicular fluid were determined by ELISA, as described [18], and only biological samples that met the following criteria were selected: E2/P4 concentration ratio > 1 for Dev (n = 3) and PostDev (n = 3), and < 1 for DevF2 (n = 3) and PreOv (n = 3) [19].

RNA was extracted from follicular cells using TRIzol reagent (Invitrogen, Waltham, MA, USA) according to the manufacturer's instructions. RNA sequencing was performed using the TruSeq RNA Sample Preparation Kit (Illumina, San Diego, CA, USA) following manufacturer's instructions, at the Centro de Genômica Funcional ESALQ/USP. Briefly, 2 µg of total RNA from each cells sample was used for library preparation. The RNA concentration and purity were assessed using NanoDropTM (Thermos Scientific, Waltham, MA, USA) and integrity was assessed by Bioanalyzer (Agilent, Santa Clara, CA, USA). The mRNA was enriched from total RNA using oligo dT magnetic beads, poly(A) RNA was fragmented, and cDNA synthesized. Thereafter, final cDNA repair was performed. The 3' ends were adenylated, and universal barcode adapters were ligated to the cDNA fragments to perform solid phase PCR and produce the sequencing library; the latter was evaluated and quantified using an Agilent 2100 Bioanalyzer and quantitative PCR with a KAPA Library Quantification kit (KAPA Biosystems, Foster City, CA, USA). Finally, libraries were pooled to perform multiplexing sequencing using HiSeq SBS v4 High Output Kit. Cluster generation and sequencing were performed on the Illumina HiSeq 2500, with 2 × 125 bp pair-end reads produced.

### Indexing and genome alignment

The reference genome (*Bos taurus* ARS-UCD1.2) was indexed, and alignment of the reads to the indexed genome was performed using STAR [20]. The number of reads per gene was determined using the quantMode and twopassMode configurations of the STAR package.

### Statistics and bioinformatic analyses

The statistical power of this experimental design, calculated in RNA-Seq | Power analysis software (https://rodrigo-arcoverde.shinyapps.io/rnaseq_power_calc/), was 0.87. This calculation was performed using the following parameters: Sequencing depth: 87; Sample size: 15; Coefficient of variation: 0.4, Effect: 1.6; and alpha: 0.05.

After obtaining reads, groups were analyzed using the DESeq2 program script, an R/Bioconductor package in the R programming language (v 4.0.2) [21]. Data were normalized, and transcripts with samples having an average normalized count < 5 were removed. The Benjamini FDR correction for multiple testing was applied to the statistical test to avoid Type I errors. Genes were considered differentially expressed genes (DEGs) when |log2fold change| was > 1.5 and FDR was < 0.05. A stepwise transcriptome comparison between follicles was conducted (DevF2 vs. PreDev; Dev vs. DevF2; PostDev vs. Dev and PreOv vs. PostDev). However, a comparison of the PreDev vs. Dev transcriptome was not included in the analysis due to the presence of only one DEG. Conserved and non-conserved DEGs in various follicles were analyzed using an Upset diagram. Heatmap and principal component analysis (PCA) were performed using Heatmap3 [22] and plotPCA [23] packages, respectively, in the R programming language (Version 4.0.2). Correlation analyses were used to evaluate gene expression variation among replicates within the same follicular phase. There were strong positive correlations, indicating a high level of homogeneity among replicates (Additional file 1).

### Gene ontology (GO) and gene set enrichment analysis (GSEA)

Functional enrichment analysis was performed using g:Profiler [24]. Only genes with annotations were considered, and the statistical test employed was the Benjamini–Hochberg FDR with a significance threshold of p < 0.05. GO terms were organized and visualized using the GOplot package [25]. GSEA, using GSEA software [26], was conducted only on DEGs of PreOv follicles. This analysis was performed with a pre-ranked configuration, adjusted for 1000 permutations, using the weighted enrichment statistic and a minimum and maximum gene size of 15 and 500, respectively. The established p-value and FDR Q threshold were p < 0.05 and p < 0.1, respectively. Similarity was determined using the overlap coefficient with a combined constant of 0.5. However, Dev, DevF2, and PostDev follicles were not included in the results, as the prior pathway analysis had FDR values with p > 0.05.

### Protein–protein interaction (PPI) and Hub genes

A PPI network was generated for each follicle using String software, Version 11.5 [27]. Network settings included evidence-based node communication and interaction sources (text mining, experiments, databases, co-expression), with an interaction score of 0.900. Once the network was created, it was exported to Cytoscape [28], and Hub DEGs were determined using the cytoHubba package [29]. Topological methods including Maximal Clique Centrality (MCC), Density of Maximum Neighborhood Component (DMNC), Maximum Neighborhood Component (MNC), degree, Edge Percolated Component (EPC), bottleneck (BN), eccentricity, closeness, radiality, betweenness, stress, and clustering coefficient were considered for each PPI figure. The top 20 DEGs highlighted by each method were selected, and after intersection analysis, DEGs identified with at least five methods were defined as Hub DEGs. GO analysis of the Hub DEGs in the PostDev and PreOv follicles was performed using the GOplot package.

### Transcription factors

Transcription factors were identified by comparison of a DEGs list and the transcription factor database [30].

### Deconvolution and KEGG pathway analysis

Cellular heterogenicity was predicted in DevF2, Dev, PostDev, and PreOv follicles by comparing a list of paralogous genes with data published in the murine cyclic ovary atlas (SCP1914, https://osf.io/924fz/). The analysis was performed using CIBERSORT [31]. The signature matrix was created using data published in the murine cyclic ovary atlas. The parameters setting was considered as default. KEGG pathways analysis was performed using the Pathview tool [32]; for input, the DEGs list was designed from PostDev and PreOv follicles. However, DevF2 and Dev follicles were not considered for this analysis, due to lower gene numbers.

## Results

### Follicular cells transcriptome

**Fig. 1:**
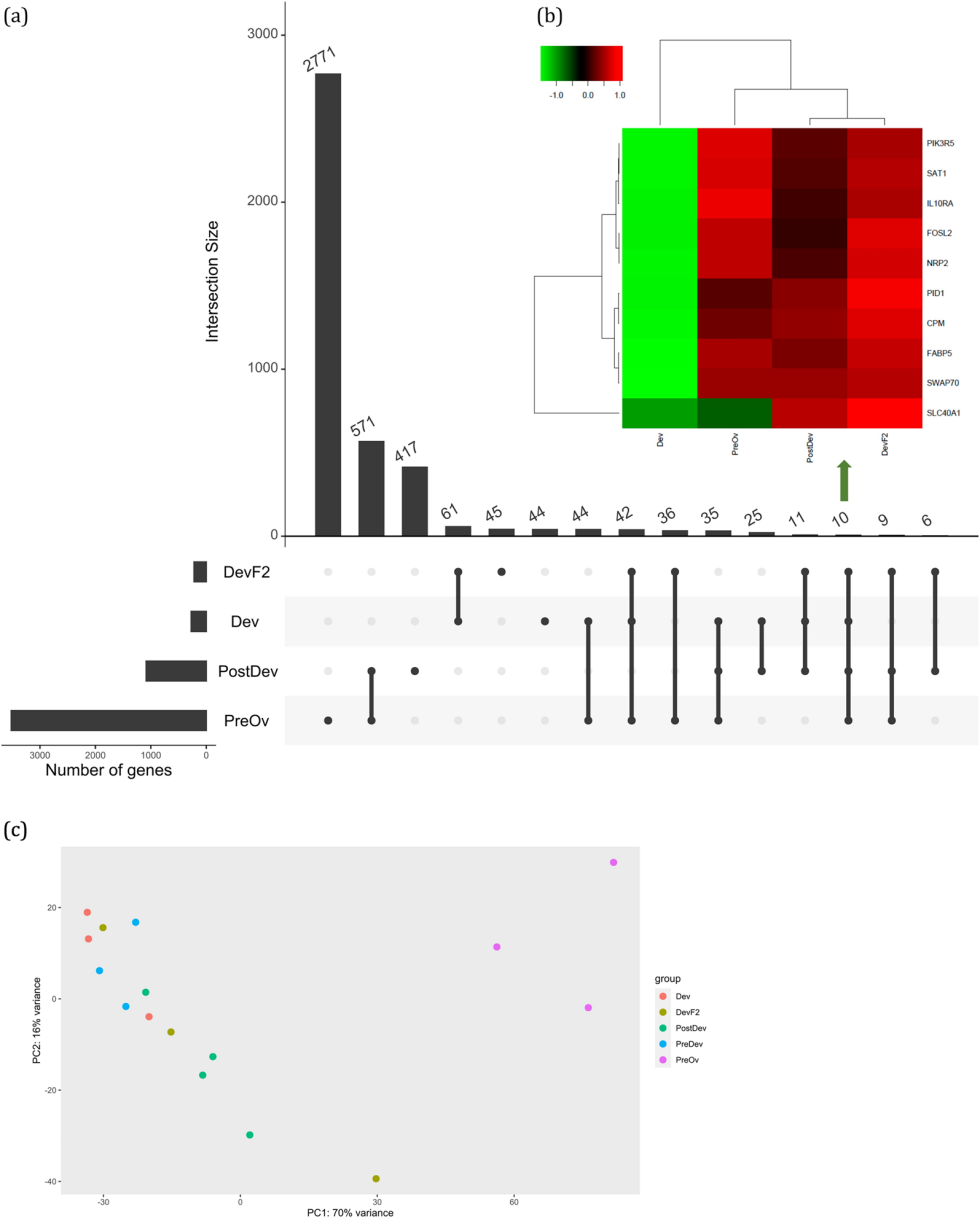
**a** Number and intersection DEGs in follicular cells of Dev, DevF2, PostDev and PreOv bovine ovarian follicles. **b** Heatmap showing upregulated (red) and downregulated (green) DEGs conserved in follicular cells of Dev, DevF2, PostDev and PreOv follicles. **c** PCA plot group by Dev, DevF2, PostDev and PreOv follicle stage. Each stage is represented by its own color

The RNA Integrity Number (RIN) of the samples analyzed ranged from 6.7 to 9.4. From the 27,270 genes identified, 1805 (6.6%) were differentially expressed. The total number of reads per sample is in Additional file 1. As follicles developed, there was an increase in number of DEGs. In DevF2, Dev, PostDev, and PreOv follicles, there were 219 (198 upregulated and 21 downregulated), 271 (42 upregulated and 229 downregulated), 1081 (1043 upregulated and 38 downregulated), and 3508 (2214 up and 1294 down) DEGs, respectively (Fig. 1A).

The DEGs conserved in all follicular stages were *PIK3R5*, *SAT1*, *IL10RA*, *FOSL2*, *NRP2*, *PID1*, *CPM*, *FABP5*, *SWAP70*, and *SLC40A1*; in the Dev follicle, all these DEGs expressed lower values than the average. In contrast, in the PreOv follicle, only DEG *SLC40A1* had values lower than the average (Fig. 1B).

Differentiation of follicles considering DEGs was evaluated by principal component analysis (PCA). There was a clear separation of samples belonging to the PreOv category; however, DevF2, Dev, and PostDev follicles were not distinct (Fig. 1C). Additional PCA information between follicles is in Additional file 1. Additionally, intrafollicular and interfollicular variation was evaluated with collaboration and sample-to-sample distance analysis (Additional file 1). Additional file 1 file has all upregulated and downregulated DEGs for each follicle.

### Gene ontology (GO)

**Fig. 2:**
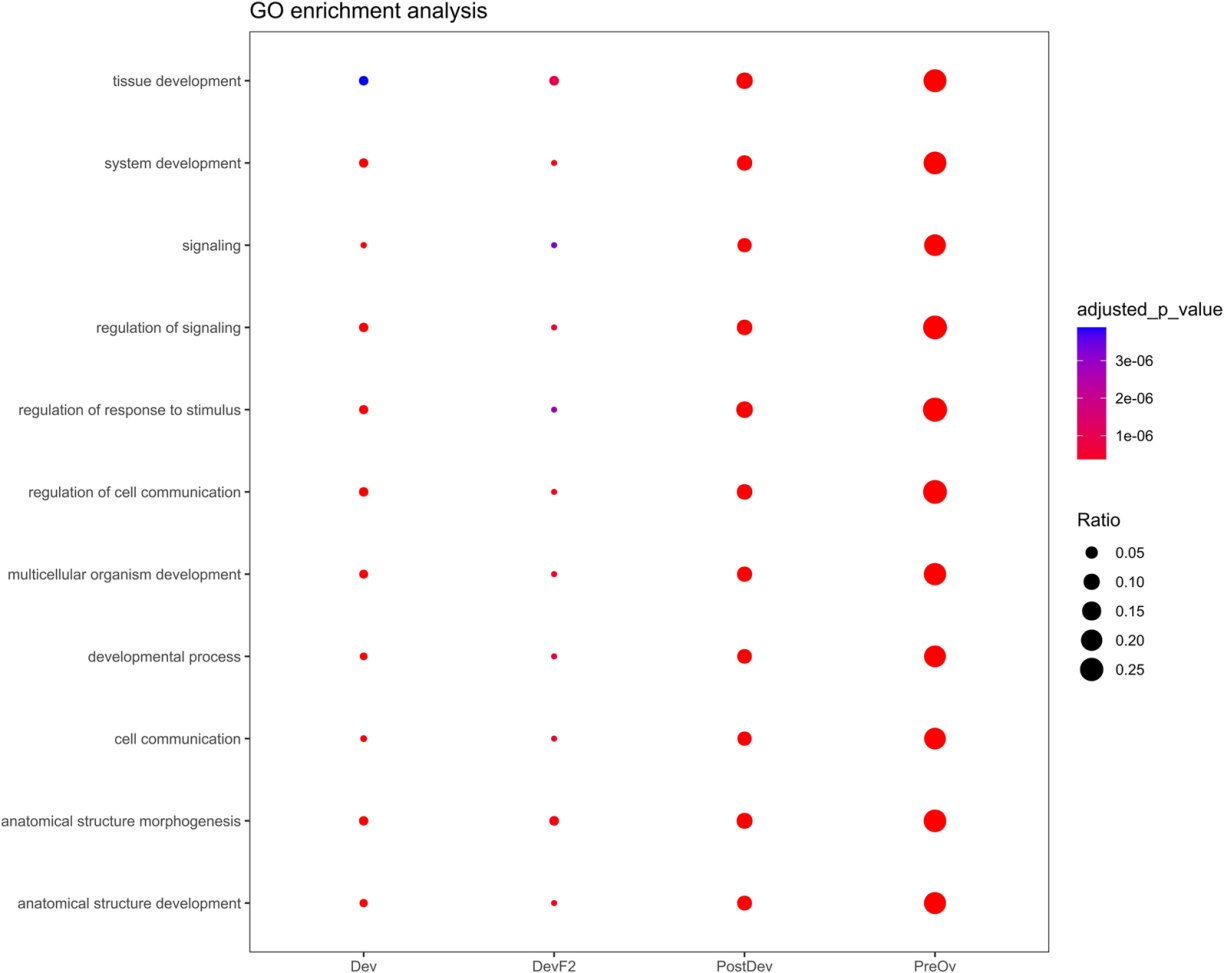
Gene ontology enrichment analysis of follicular cells of Dev, DevF2, PostDev and PreOv bovine ovarian follicles. The ontology terms used were selected to be conserved among follicle stages. The circle size indicates the ratio between input DEGs and ontology term genes. Significance is shown as higher (purple) and lower (red)

Functional enrichment of DevF2, Dev, PostDev, and PreOv follicles was performed with g:Profiler and illustrated with GOCircle. Functional annotations related to biological processes, molecular functions, and cellular components conserved between follicles are shown in Fig. 2. The terms tissue development, signaling, and regulation of response to stimulus had greater significance; however, the ratio of genes included in the ontological term versus genes identified in the study was higher in the PreOv follicle followed by the PostDev follicle (Fig. 2). Additional file 1 includes all GO terms associated with each follicle stage.

### Gene set enrichment (GSEA)

**Fig. 3:**
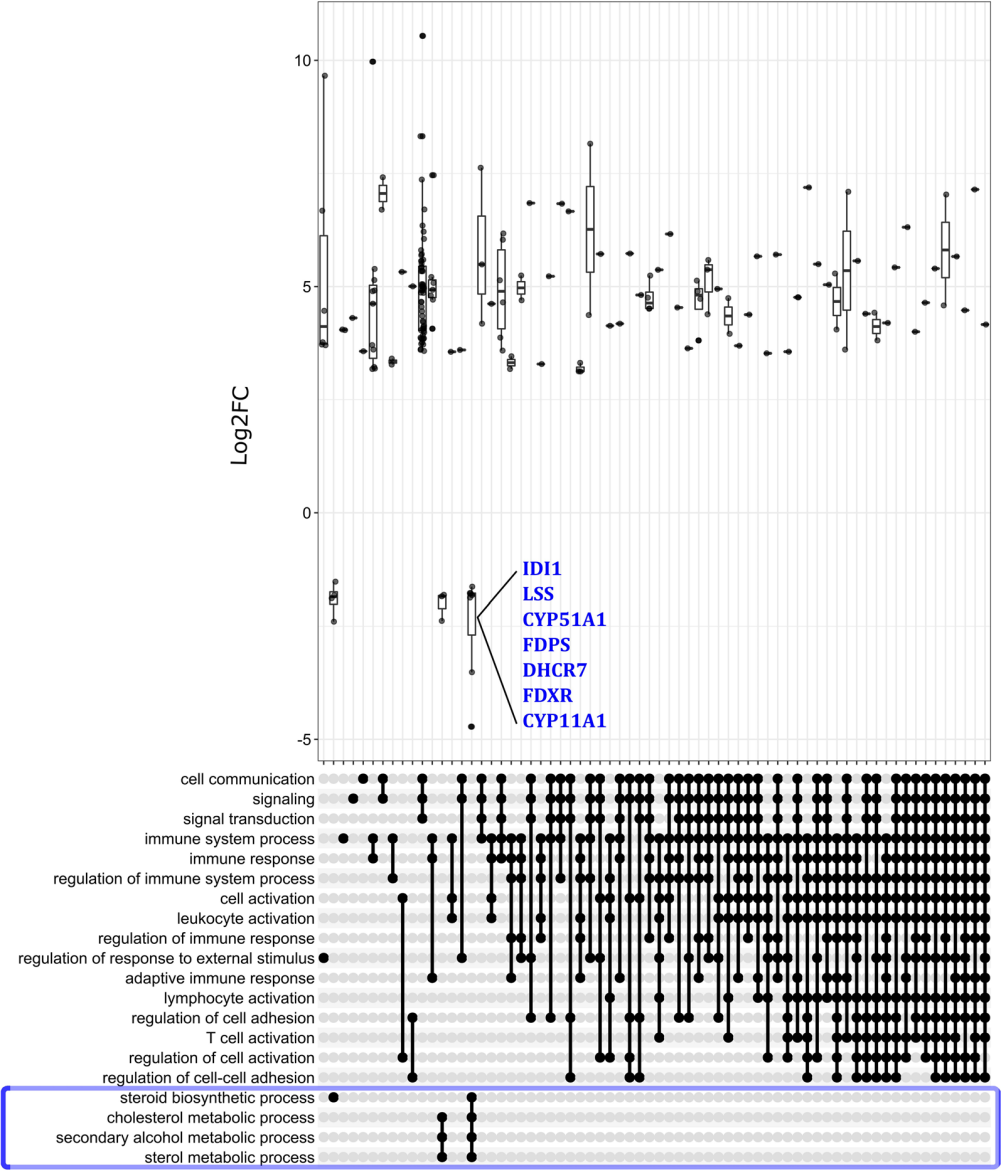
Gene set enrichment analysis (GSEA) of follicular cells of PreOv bovine ovarian follicles. Top 20 pathways are shown in rows. Points in the bottom panel indicate gene intersection among pathways. The top panel indicates the directionality of DEGs conserved among pathways. The blue frame highlights pathways downregulated in PreOv follicle cells. DEGs associated with downregulated pathways are in blue

Gene pool enrichment included the PreOv follicle and was performed considering the pre-ranked configuration of the GSEA software. DevF2, Dev, and PostDev follicles were not included because they did not have enough DEGs. However, DEGs *IDI1*, *LSS*, *CYP51A1*, *FDPS*, *DHCR7*, *FDXR*, and *CYP11A1* were downregulated and conserved in the ontological terms steroid biosynthetic process, cholesterol metabolic process, secondary alcohol metabolic process, and sterol metabolic process; therefore, these results exhibited downregulation of pathways associated with steroidogenesis (Fig. 3). Based on GSEA analysis, terms mostly related to immunity were upregulated in the PreOv follicle (Fig. 3).

### Protein–protein interaction (IPP) and Hub genes

**Fig. 4:**
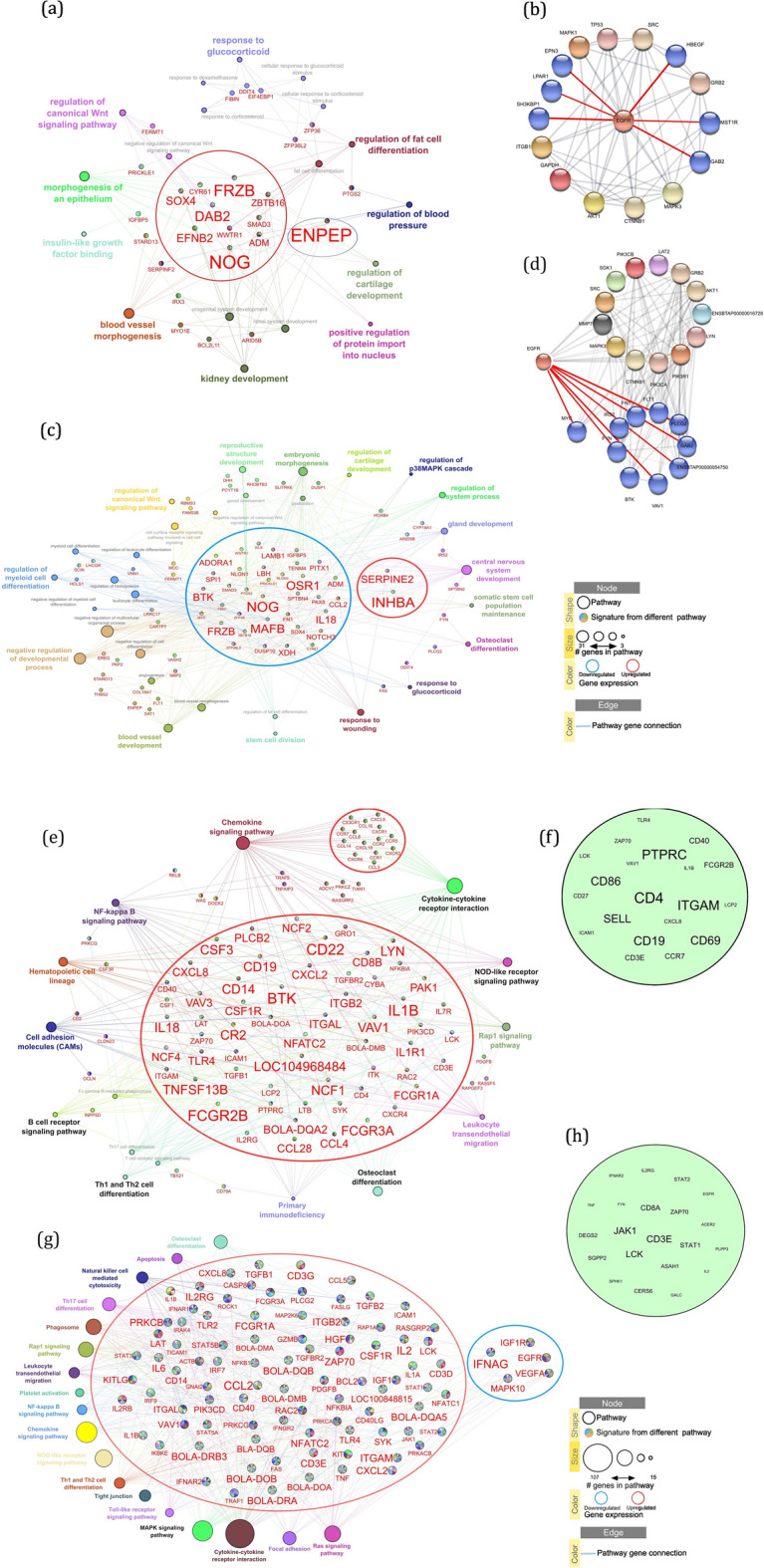
Network pathway analysis of **a** DevF2, **c** Dev, **e** PostDev, and **g** PreOv follicles cells from bovine ovarian follicles. In the center of each network are DEGs that were identified at the least in three pathways. The size of DEGs name indicates the expression level. Ellipses around DEGs indicate upregulated (red) or downregulated (blue) direction. Pathways are represented as circles and various colors. Major circles indicate a pathway with a higher number of DEGs. Hub DEGs of **b** DevF2, **d** Dev, **f** PostDev, and **h** PreOv follicles. Blue nodes signal a connection with EGFR Hub gene (**b**, **d**)

The main findings inherent to DevF2 and Dev follicles after analyzing two types of networks, one created with ClueGo and the other with String, are shown in Fig. 4a–d, respectively. The network elaborated by ClueGo (Fig. 4a) gathered DEGs associated with distinct ontological terms and that presented a central activity in modulation of molecular processes; this approach allowed demonstrating that the upregulated DEGs *SOX4*, *FRZB*, *ZBTB16*, *DAB2*, *NOG*, *SMAD3*, *ADM*, and *WWTR1* had central activities in the DevF2 follicle (Fig. 4a). In the Dev follicle, the DEGs mentioned above were also identified, although the directionality of expression changed to a downregulation profile (Fig. 4c). Furthermore, other DEGs such as *MAFB*, *IL18*, and *FBN1* were downregulated. In addition, *SERPINE2* and *INHBA* DEGs, which were upregulated, exerted relevant actions in several molecular pathways (Fig. 4c). In both follicles (DevF2 and Dev), analysis of the IPP network indicated that several nodes represented by proteins interacted with the *EGFR* protein (Fig. 4b and d).

In PostDev and PreOv follicles, identification of DEGs with central action was determined, including identification of Hub DEGs in the network analysis (ClueGo and String) with subsequent evaluation of the intersection. The top 20 Hub DEGs and the network with the DEGs and associated molecular pathways of PostDev and PreOv follicles are shown (Fig. 4e–h, respectively). Based on the 12 topological methods and intersection analysis, the top 20 Hub DEGs in the PostDev follicle were *PTPRC*, *TLR4*, *ZAP70*, *LCK*, *CD40*, *VAV1*, *FCGR2B*, *CD86*, *CD27*, *CD4*, *ITGAM*, *LCP2*, *SELL*, *CXCL8*, *ICAM1*, *CD19*, *CD69*, *CD3E*, and *CCR7* (Fig. 4f), whereas in the PreOv follicle they were *IL2RG*, *IFNAR2*, *STAT2*, *EGFR*, *TNF*, *FYN*, *CD8A*, *ZAP70*, *ACER2*, *DEGS2*, *JAK1*, *CD3E*, *STAT1*, *PLPP3*, *SGPP2*, *LCK*, *ASAH1*, *IL2*, *SPHK1*, *CERS6*, and *GALC* (Fig. 4h). In both the PostDev follicle (Fig. 4e) and the PreOv follicle (Fig. 4g), DEGs were associated with pathways that stimulate immunity (e.g., chemokine signaling and cytokine-cytokine receptor interaction), pathways that in turn had a higher number of DEGs. Additionally, in the ClueGo network approach, the PreOv follicle had DEGs *IGFR1*, *EGFR*, *IFNAG*, *VEGFA*, and *MAPK10* downregulated (Fig. 4g).

### Transcription factors

**Fig. 5:**
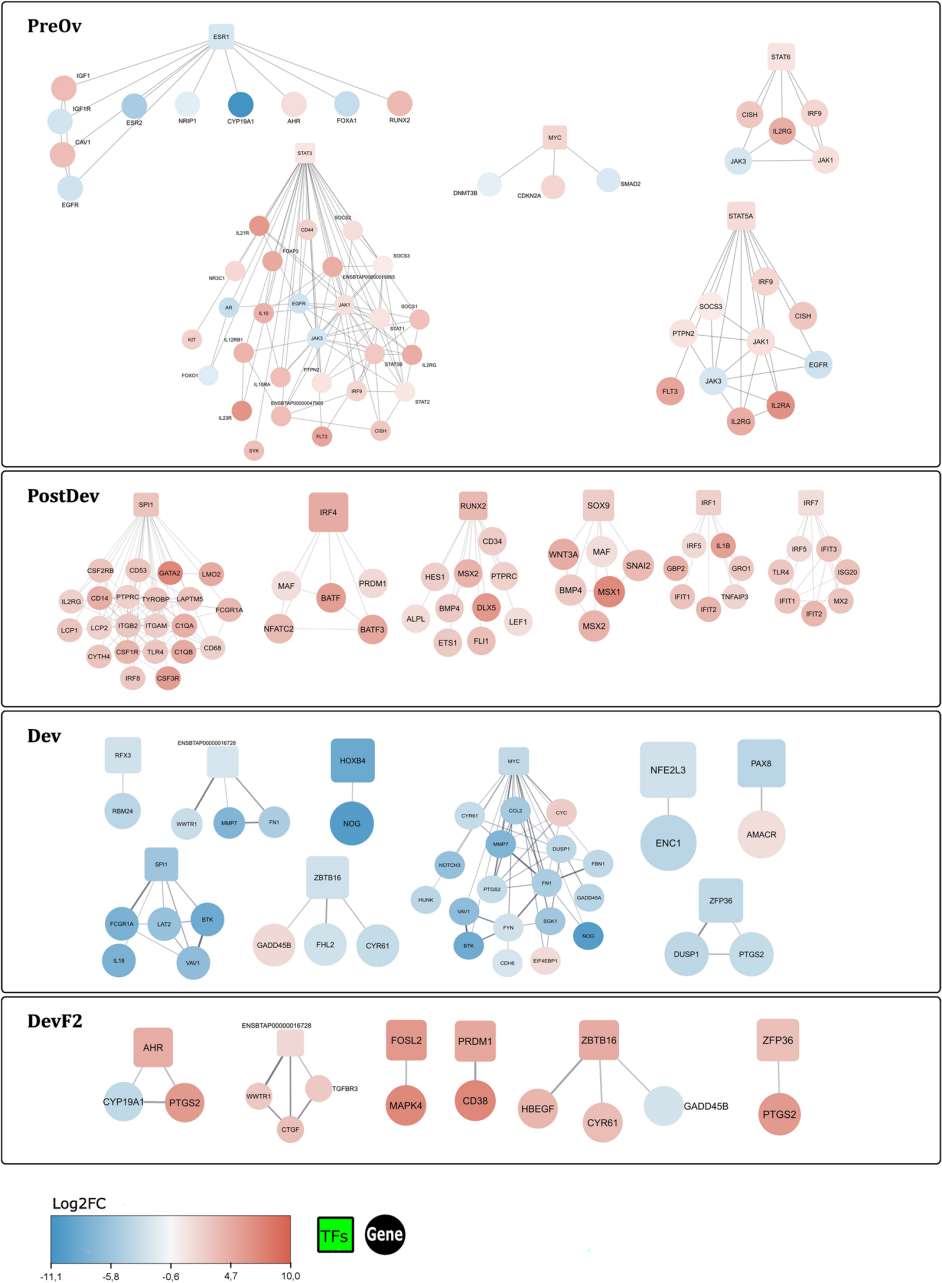
Transcription factors and its targets in Dev, DevF2, PostDev and PreOv follicular cells from bovine ovarian follicles. Expression gene direction is represented by blue (downregulated) or red (upregulated)

Considering the DEGs, all transcription factors identified in DevF2 follicles were upregulated (*AHR*, *FOSL2*, *PRDM1*, *ZBTB16*, *ZFP36*, and *ENSBTA00000016728*), whereas in Dev follicles, all were downregulated (*RFX*, *ENSBTA00000016728*, *SPI1*, *ZBTB16*, *MYC*, *HOXB4*, *NFE2L3*, *PAX8*, and *ZFP36*). The *MYC* transcription factor interacted with 18 nodes (Fig. 5). Six transcription factors were detected in PostDev follicles (*SPI1*, *IRF4*, *RUNX2*, *SOX9*, *IRF1*, and *IRF7*), all of which were upregulated. The PostDev follicle transcription factor with the highest node connection was *SPI1* (Fig. 5). Finally, PreOv follicles had four upregulated (*STAT3*, *MYC*, *STAT6*, and *STAT5A*) transcription factors but only one downregulated (*ESR1*). The *STAT3* transcription factor connected the highest number of nodes, 28 in total (Fig. 5).

### Deconvolution and KEGG pathway analysis

**Fig. 6:**
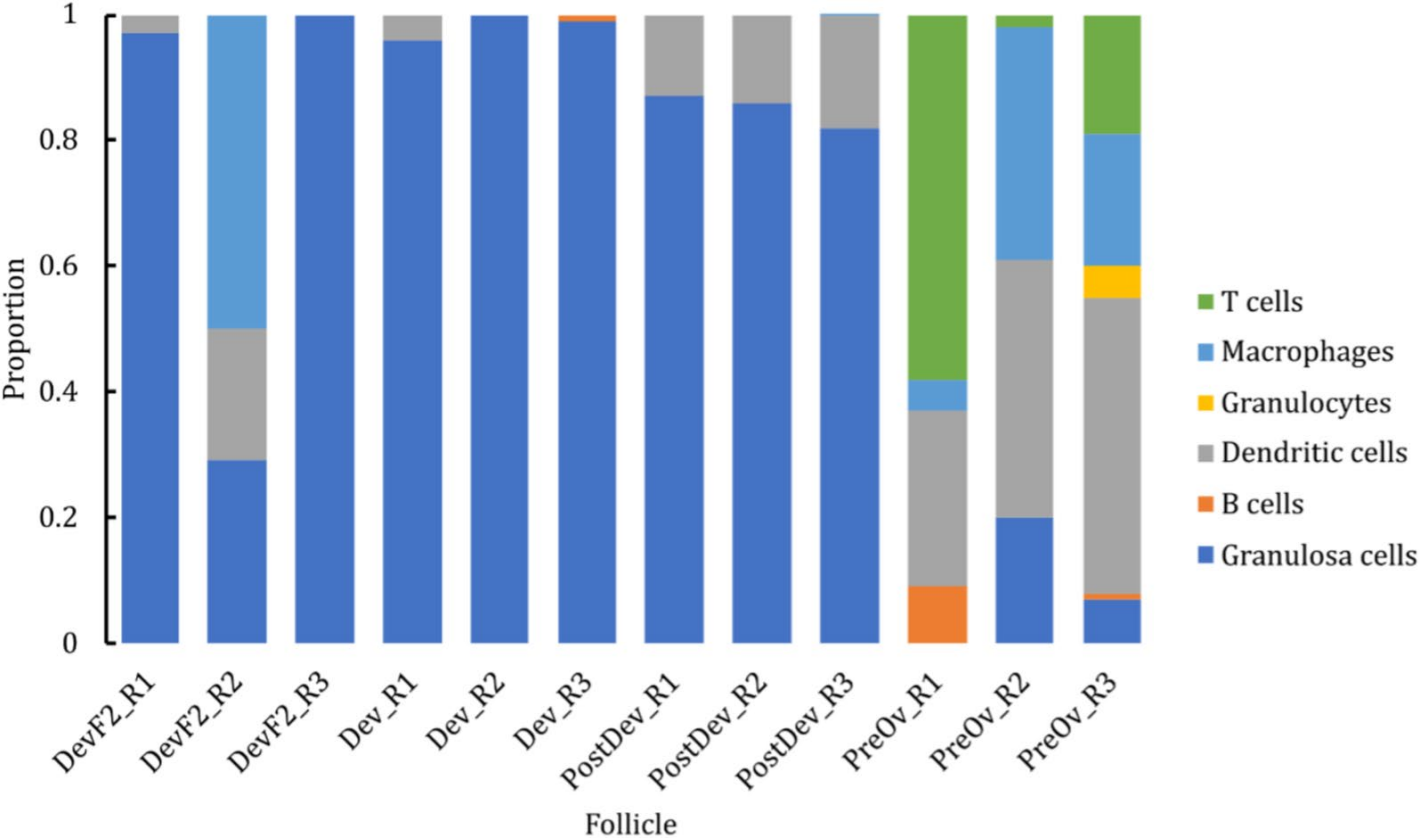
Proportions of follicular cells obtained from deconvolution analysis in each replicate of Dev, DevF2, PostDev, and PreOv follicles from bovine ovaries

**Fig. 7:**
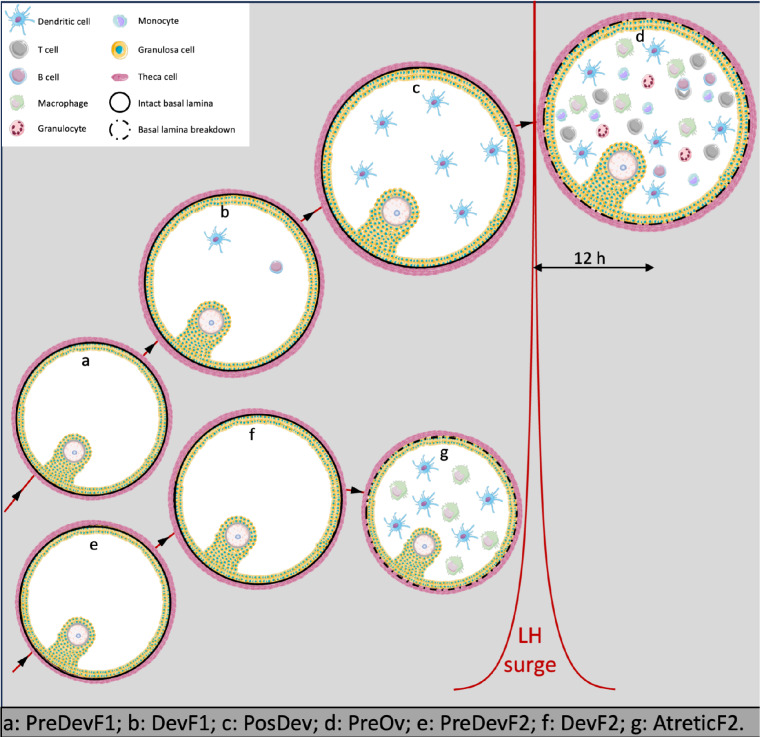
Theoretical model of changes of cell population during follicular development in Holstein cows

Cellular composition of DevF2, Dev, PostDev, and PreOv follicles was predicted by comparing the list of paralogous genes with data in the murine cyclic ovary atlas (SCP1914, https://osf.io/924fz/). Based on the predicted data, DevF2, Dev, and PostDev follicles had different proportions (p < 0.05) of GCs (Fig. 6). In PreOv follicles, the proportion of GCs was lower; however, there was a higher proportion (p < 0.05) of macrophages, B cells, T cells, and dendritic cells (Fig. 6). Based on these findings, we developed a theoretical model to describe the dynamics of the cell population in bovine ovarian follicles throughout their development (Fig. 7).

**Fig. 8:**
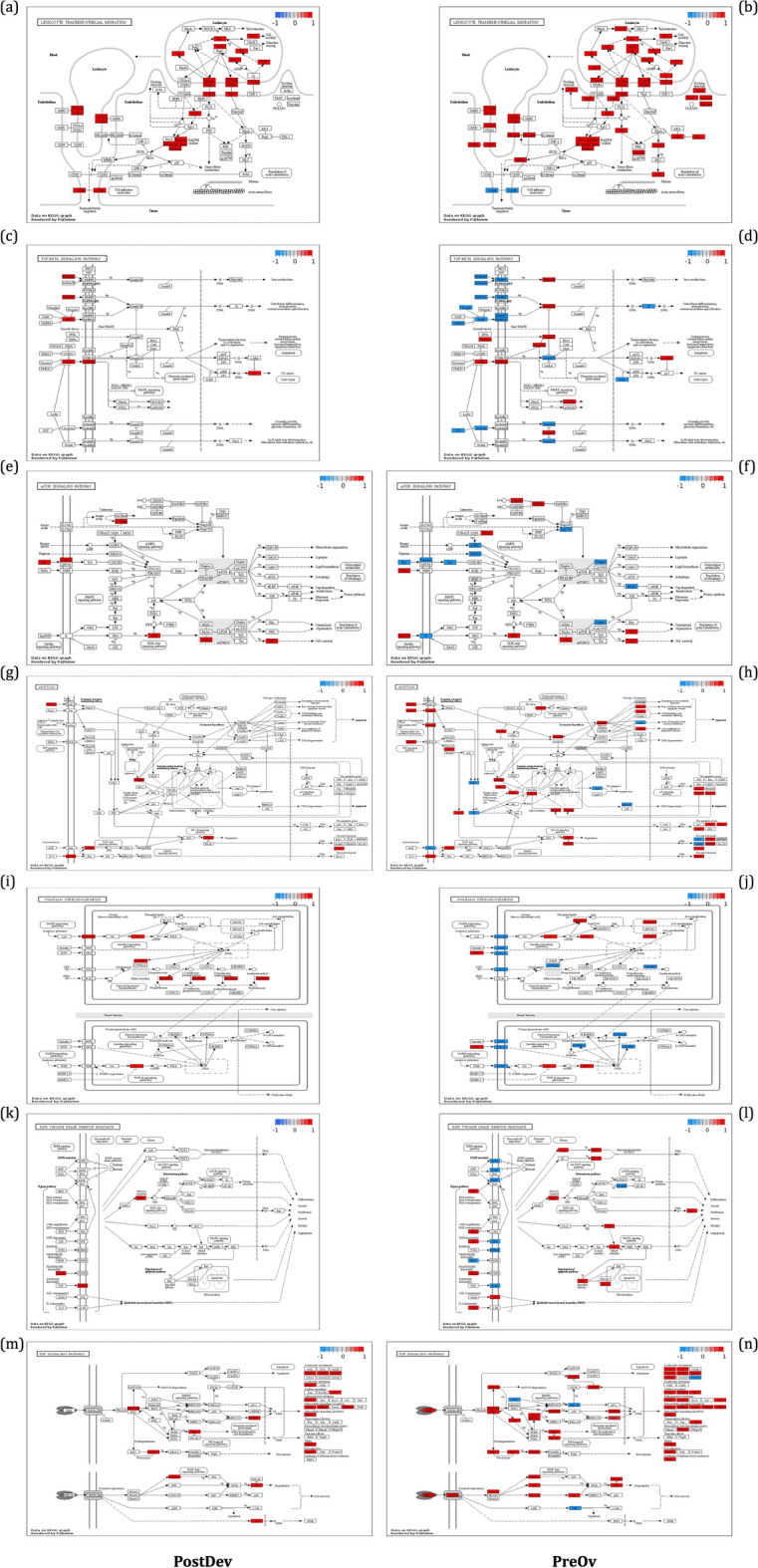
Pathview visualization of KEGG pathways from PostDev and PreOv follicles. Upregulated and downregulated genes are indicated by red and blue colors, respectively

KEGG pathway analysis was performed on PostDev and PreOv follicles. In the PreOv follicle, the TGF-B pathway (Fig. 8d), mTOR pathway (Fig. 8f), steroidogenesis pathway (Fig. 8j), and EGFR pathway (Fig. 8l) exhibited downregulated gene expression. Furthermore, reduced transcript levels of genes *OCLN* and *CLDN23*, responsible for producing proteins that facilitate cell adhesion, were observed in the transendothelial leukocyte migration pathway (Fig. 8b). Additionally, the apoptosis pathway (Fig. 8h) and TNF pathway (Fig. 8n) had more differentially expressed genes than were present in PostDev follicles.

## Discussion

Ovarian follicular development in cattle is regulated by endocrine, paracrine, and autocrine stimuli [3]. The GCs, stimulated by these agents, undergo complex structural and functional changes as follicles develop. At deviation, a critical feature of the future dominant follicle is its greater capacity for E2 production compared to the second largest follicle of the wave. In this study, only DevF2 follicles with an E2/P4 ratio < 1 were selected for the RNAseq assay, to ensure representativeness of this class [19].

The comparison of DevF2 vs. PreDev by the ClueGo and cytoHubba analysis of the DEGs revealed super-expressed genes in DevF2 involved with the WNT and transforming growth factor-β (TGF-β) superfamily pathways. The TGF-β superfamily includes bone morphogenetic proteins (BMPs), activin, antimüllerian hormone (AMH), and TGF-β [33]. In our study, the DevF2 upregulated DEGs identified closely related to TGF-β were *NOG*, *SMAD3*, *ZBTB16*, and *DAB2*. The NOG protein binds to several BMPs (BMP2, BMP4, BMP5, BMP6, BMP7, BMP14) [34–36] and GDF5 protein [37] receptors, blocking their functions related to cell proliferation, differentiation, motility, and extracellular matrix production [33]. So, NOG probably has a critical role in GCs, resulting in loss of their viability and induction of follicular atresia.

Canonical and non-canonical TGF-β pathways are mainly involved with cell death and cell survival, respectively. Through the canonical pathway, the TGF-β family members bind to their receptors in the cell membrane, promoting phosphorylation of SMAD2/3, which binds to SMAD4, forming a complex that enters the nucleus and recruits cofactors that control expression of genes related to proliferation, cell differentiation, or apoptosis [38, 39]. Overexpression of SMAD3 in DevF2 also seemed to be related to GCs functional impairment and follicular atresia, as observed in GCs of women with polycystic ovaries, where its overexpression triggers apoptosis of GCs [40].

It was reported that *DAB2* is upregulated in GCs of atretic bovine follicles [41]. DAB2 protein inhibits the non-canonical pathway of TGF-β, which promotes cell survival and proliferation, and activates the canonical pathway that suppresses cell growth and induces cell death [42]. Overexpression of *DAB2* in our study reinforced its involvement as a potential mechanism contributing to follicular atresia. Notably, this effect was more pronounced in DevF2_R2, with a higher degree of degeneration characterized by massive infiltration of immune cells (Fig. 6).

*ZBTB16* encodes a zinc finger transcription factor involved in cell cycle progression [43]. This gene is associated with development of polycystic ovaries in obese women [44], antiproliferative activity in prostate cancer cells [45] and undifferentiated spermatogonia renewal [43]. It also acts as a transcription factor to *HBEGF*, which stimulates in vitro transcription of *STAR* and P4 production by human GCs [46]. *ZBTB16* is also a transcript factor for *CYR61* [30] a critical marker of tumor cell inflammation [45]. HBEGF and CYR61 proteins can be associated with increased P4 production and inflammatory response, respectively, observed in DevF2 and PreOv follicles in our study.

Genes *FRZB*, *SOX4*, and *WWTR1* were also overexpressed in DevF2. Increased expression of *SOX4* and *FRZB* transcripts is associated with activating the canonical Wnt/β-catenin pathway.

The Wnt glycoproteins bind to their cell membrane receptors complex (Lrp5/6 and FRZB) inhibiting β-catenin and/or WWTR1 phosphorylation and allowing them to be translocated into the nucleus and to activate transcription factors for target genes involved in cell proliferation and apoptosis inhibition [47–51]. These findings may represent a rescue mechanism, as the DevF2 follicle can become dominant if the Dev follicle is ablated at deviation [52].

*CYP11A1*, *CYP19A1*, *DHH*, *PAPPA*, and *NRXN2* appeared as DEGs downregulated in DevF2 follicles (Additional file 1). CYP19A1 converts androgens into E2, whereas CYP11A1 catalyzes conversion of cholesterol into pregnenolone. Therefore, our findings seem to be related to the expected decreased follicular steroid production capacity, particularly E2, an important indicator of follicular atresia [53].

Follicular atresia also seems to be linked to the observed decreased transcription of *PAPPA,* gene that encodes a metalloproteinase responsible for cleaving IGFBP4 [54]. Reduced *PAPPA* expression can lead to an accumulation of IGFBP4, which hinders stimulatory effects of insulin-like growth factors (IGFs) [55] that enable follicles to grow under low concentrations of follicle stimulating hormone (FSH), a critical feature for selection and dominance [3].

*DHH* is part of the Hedgehog gene family, which also includes Sonic (*SHH*), Indian (*IHH*), and Desert (*DHH*) genes. Interestingly, knockout of both *IHH* and *DHH* genes in mice decreased steroidogenesis and caused infertility [56]. Furthermore, decreased expression of the *DHH* gene can be involved in decreased steroidogenesis by the DevF2 follicle.

Our study was apparently the first to report decreased expression of the *NRXN2* in DevF2 follicles at the expected time of deviation, a gene known to be involved in transmission of nerve signals and hypogonadism [57].

Selection of the dominant follicle during a follicular wave is manifested by continued growth of the largest follicle but a decreased growth rate of the second largest. At 12 h before diameter deviation, the future dominant follicle has a series of gradual changes, including increased granulosa LH receptors and E2 production [3]. In Dev follicles, the Adrenomedullin (*ADM*) gene was downregulated. *ADM* encodes a protein primarily involved in vasodilation [58]. ADM protein is known for its pleiotropic effects, impacting various processes (e.g., vascular regulation, angiogenesis and cell development). The ADM effects involve its interaction with the calcitonin receptor-like receptor (CLR) heterodimer and with the receptor activity-modifying proteins, specifically RAMP2 and RAMP3, that couple with ADM2 and 3, respectively. This interaction increases intracellular concentrations of cAMP [59] and activation of stimulus-dependent pathways including PI3K, PKA, cGMP, and MAPK [60].

In rat granulosa [61] and Sertoli cell [62] cultures, exogenous FSH decreased *ADM* gene expression. Moreover, in GCs, this effect was potentiated when hCG was added to culture medium [61]. In our study, there was downregulation of *ADM* and upregulation of *CYP19A1* in developing follicles with a higher E2:P4 ratio. Therefore, we inferred a potentially crucial paracrine role for ADM in hindering steroidogenesis and deviation. To the best of our knowledge, this is the first report of in vivo involvement of ADM in bovine follicular deviation.

In DevF2 follicles, there was a higher transcript level of the *IL18* gene, which codifies a pro-inflammatory cytokine with a pivotal role in host defense by regulating genes associated with inflammation, infection, and malignancy [62]. In women with polycystic ovary syndrome (PCOS), their GCs and follicular fluid have higher levels of *IL18* transcript and IL18 protein, respectively [63], implying involvement of this agent in the pathogenesis of these ovarian dysfunctions. Additionally, in vitro supplementation of bovine theca cells with *IL18* upregulated key steroidogenic-related genes and increased androstenedione secretion (*CYP17A1*, *CYP11A1*) [64].

As observed for *IL18*, a similar expression pattern was evident for the *MAPF* gene, a known marker of myeloid cells [65], in Dev2 follicles. These transcripts were notably higher in replica 2 of DevF2 follicles that had a massive invasion of myeloid cells (Fig. 6). Therefore, upregulation of *MAPF* and *IL18* are potential markers of follicular myeloid cell invasion, inflammation, and atresia.

Regarding Dev follicles, an important discovery in our study was downregulation of *FBN1*. When *BMP15* silences this gene, it increases cell proliferation and prevents apoptosis in porcine cumulus cells [66]. Therefore, decreased expression of *FBN1* gene in GCs of Dev follicles may represent a mechanism for cell survival and development [67].

After clueGO and PPI analyses of Dev follicles, two upregulated genes, *INHBA* and *SERPINE2*, were detected participating in more than three ontological pathways. The *INHBA* encodes the inhibin βA subunit, which combines with the α subunit to form Inhibin A; this suppresses FSH production and secretion, thereby inducing atresia of subordinate ovarian follicles [68]. Inhibin A also enhances viability, mitochondrial membrane integrity, and proliferation of GCs while inhibiting apoptosis. Protective effects of inhibin are associated with downregulation of *BAX* and *Caspase 3* [69].

The *SERPINE2* gene encodes a protein that belongs to the Serpin superfamily, molecules responsible for inhibiting plasminogen activators (PAs), a group of proteases involved in various processes during follicular development, particularly in follicle rupture during ovulation [70]. Inhibition of PAs by serpins has a crucial role in preventing apoptosis in GCs in hamsters and cattle [71, 72]. Hence, upregulation of *SERPINE2* in Dev follicles in this study could be a vital mechanism for safeguarding them by inhibiting apoptosis and preventing early follicular rupture. Therefore*, SERPINE2,* and the previously mentioned Dev follicle that retained its transcripts (*PAPPA*, *CYP19A1*, and *CYP11A1*) appeared in our study as critical indicators of follicular health and steroidogenic capacity.

Identification of 20 hub genes in PostDev follicles indicates significant transcriptional activity related to various immune processes, including neutrophil chemotaxis (*CXCL8*) [73], endothelial transmigration and adhesion of neutrophils and monocytes (*ITGAM*, *SELL*, and *ICAM1*) [74–76], T-cell adhesion and migration to inflamed tissues [73], recognition of pathogen-associated molecular patterns (PAMPs), inflammatory cytokine production, and activation of innate immunity mediated by myeloid cells (*TLR4*) [77] antigen recognition in T-cells (*CD4*, *LCP2*, and *CD3E*) [73] and B-cells (*PTPRC*) [78], development and activation of T and B-cells (*ZAP70*, *CD86*, *LCP2*, *VAV1*, *CD27*, and *LCK*) [73, 79], autoimmune responses and maturation and apoptosis of T-cells (*LCK*) [80], and activation of antigen-presenting cells (*CD40*) [81]. These findings highlighted an early invasion and perhaps a critical role of immune cells in the follicular environment and in follicular development, even before the LH peak.

In our investigation of hub genes in PreOv follicles following the GnRH-induced LH peak, we identified two critical molecular mechanisms: production of sphingolipid metabolite sphingosine-1-phosphate (S1P) and the immune response.

Activation of S1P production machinery was characterized by upregulation of genes *GALC*, *CERS6*, *ASH1*, and *SPHK1*, and downregulation of the *SGPP2* gene, involved in S1P degradation. S1P acts as a second messenger in cell signaling but is primarily extracellularly exported by proteins as major facilitator superfamily transporter 2b (MFSD2b) [82] and protein spinster homolog 3 (SPNS2) [83], which were both upregulated in preovulatory follicles.

S1P exerts its extracellular effects by signaling through five specific G protein-coupled receptors (S1PR1–5). Furthermore, S1P, acting through S1PR1 and S1PR3, has a crucial role in various processes, including vasodilation and vascular maturation [84]. In human GCs, it increases prostaglandin E2 synthesis, induces P4 production [85], and protects these cells from H2O2-induced apoptosis [86]. Additionally, binding to S1PR1, S1PR3 [87–89] and S1PR4 [90] induces lymphocyte trafficking, migration, and differentiation. Based on our findings and previous discoveries, S1P probably has essential roles in preovulatory follicles, enhancing immune cell migration and promoting vascular development, exerting antiapoptotic effects on GCs, and stimulating P4 production, all critical steps for luteogenesis.

Additionally, SPHK1 protein and S1P are crucial in TNF signaling and the NF-kappa-B activation pathway, which is important in inflammatory, antiapoptotic, and immune processes [91, 92]. TNF, a cytokine mainly secreted by macrophages, is involved in regulating a wide spectrum of biological processes including inflammation, cell proliferation, differentiation, apoptosis, lipid metabolism, and coagulation [93], all key processes in ovulation.

As observed in PostDev follicles, several hub genes that were identified in preovulatory follicles were involved in antigen recognition in T-cells (*CD3E* and *CD8A*) [73, 93], immune cell to cell integration (*CD8A*) [93], development, activation (*ZAP70*) [73, 79], and proliferation (*IL2* and *IL2RG*) [93] of T and B-cells, apoptosis, maturation, and activation of T-cells, as well as autoimmune responses (*LCK*) [80].

Additionally, in PreOv follicles, we also detected hub genes involved in several functions of cell downstream signaling pathways, including cell receptors (EGFR), interferon-alpha/beta receptor beta chain (IFNAR2), and membrane-associated tyrosine kinases (FYN, JAK1) [93].

In addition to hub genes, increased transcription of *MAF*, *MAFB* [65], and *PTPRC* [94] reinforced the massive myeloid cell invasion of PreOv follicles.

For apparently the first time in cattle, deconvolution analysis of the transcriptional profile of cells in follicular fluid enabled us to characterize cellular dynamics during follicular development. In the early stages, in the future dominant follicle, GCs predominate. However, as the follicle develops and reaches ovulatory capacity (~ 12 mm), migration of immune system cells (dendritic cells) occurs, albeit in small proportions. In addition, after the LH peak, there is a massive migration of immune system cells, with a predominance of dendritic cells, macrophages, and T cells, although the presence of B cells and granulocytes was also observed.

The transcriptomic profile of bovine ovarian follicular cells observed in the present study aligned closely with our laboratory's previous findings, which examined protein expression in bovine follicular fluid during various stages of follicle development. We noted a significant increase in proteins related to inflammation, immune cell chemotaxis and function—such as fibrinogen and complement components—as well as a rise in reactive oxygen species, particularly as follicles reached the final stages of development and approach ovulation [18]

## Conclusions

The transcriptional profile of cells in follicular fluid revealed a predominant presence of GCs at deviation, alongside upregulation of genes involved in viability, steroidogenesis, and apoptosis prevention. Early immune-related transcripts were also detected. In contrast, non-selected follicles had upregulation of cell death-related genes, plus some cell survival signals.

Following deviation, immune cell transcripts increased significantly in both dominant and non-selected follicles. Preovulatory follicles had strong immune activity, with upregulation of transcripts related to leukocyte chemotaxis, immune cell proliferation, and vascular changes. Therefore, we inferred there was a gradual but pronounced immune cell invasion in the follicle, likely driven by intrafollicular chemotactic signals, despite an intact basal lamina.

## Supplementary Information

Additional file 1

## Data Availability

The datasets used and/or analyzed during the current study are available from the corresponding author on reasonable request.

## Abbreviations

BN: Bottleneck
DEGs: Differentially expressed genes
Dev: Deviation largest follicle
DevF2: Deviation second-largest follicle
DMNC: Density of maximum neighborhood component
E2: Estradiol
EPC: Edge percolated component
FDR: False discovery rate
FGFs: Fibroblast growth factor family
FSH: Follicle stimulating hormone
GCs: Granulosa cells
GnRH: Gonadotropin hormone-releasing hormone
GO: Gene ontology
GSEA: Gene set enrichment analysis
IGFBPs: Insulin-like growth factors binding proteins
IGFs: Insulin-like growth factors
KEGG: Kyoto encyclopedia of genes and genomes
LHCGR: Luteinizing hormone/choriogonadotropin receptor
MCC: Maximal clique centrality
MNC: Maximum neighborhood component
P4: Progesterone
PAMPs: Pathogen-associated molecular patterns
PAs: Plasminogen activators
PCA: Principal component analysis
PCOS: Polycystic ovary syndrome
PGE2: Prostaglandin E2
PostDev: Post-deviation follicle
PPI: Protein–protein interaction
PreDevF1: Pre-deviation follicle
PreOv: Preovulatory follicle
RIN: RNA integrity number
